# Malignant pertussis in infants: factors associated with mortality in a multicenter cohort study

**DOI:** 10.1186/s13613-021-00856-y

**Published:** 2021-05-07

**Authors:** Mathilde Coquaz-Garoudet, Dominique Ploin, Robin Pouyau, Yoav Hoffmann, Julien-Frederic Baleine, Benoît Boeuf, Hugues Patural, Anne Millet, Marc Labenne, Renaud Vialet, Didier Pinquier, Marie Cotillon, Jérôme Rambaud, Etienne Javouhey

**Affiliations:** 1 Service de Réanimation et Urgences Pédiatriques, Hospices Civils de Lyon, Hôpital Femme Mère Enfant, 59 Boulevard Pinel, 69677 Bron cedex, France; 2 Laboratoire de Virologie et Pathologie Humaine—VirPath Team, Faculté de Médecine RTH Laennec, CNRS, UMR5308, INSERM U1111, Centre International de Recherche en Infectiologie (CIRI), École Normale Supérieure de Lyon, 7-11 rue Guillaume Paradin, 69372 Lyon cedex 08, France; 3 Pediatric Intensive Care Unit, Western Galilee Medical Centre, PO Box 21, 22100 Nahariya, Israel; 4 Département de Pédiatrie Néonatale et Réanimations, Hôpital Arnaud de Villeneuve, 371 Avenue du Doyen Gaston Giraud, 34295 Montpellier cedex 5, France; 5 Service de Réanimation Pédiatrique, Centre Hospitalier Universitaire Estaing, 1 Place Lucie Aubrac, 63003 Clermont Ferrand cedex 1, France; 6 Service de Réanimation Néonatale et Pédiatrique, Centre Hospitalier Universitaire de Saint-Etienne, Hôpital Nord, Pôle Mère-Enfant, 42055 Saint-Étienne cedex 2, France; 7 Service de Médecine Néonatale et Réanimation Pédiatrique, Centre Hospitalier Universitaire de Grenoble, Hôpital Couple Enfant, Boulevard de la Chantourne, 38700 La Tronche, France; 8 Service d’ Anesthésie et de Réanimation Pédiatrique, Assistance Publique-Hôpitaux de Marseille, Hôpital de La Timone, 264 Rue Saint-Pierre, 13385 Marseille cedex 5, France; 9 Service d’ Anesthésie et de Réanimation Pédiatrique, Chemin Des Bourrely, Assistance Publique-Hôpitaux de Marseille, Hôpital Nord, 13915 Marseille cedex 20, France; 10 Service de Pédiatrie Néonatale et Réanimation, Centre Hospitalier Universitaire de Rouen, Pôle Femme Mère Enfant, 1 rue de Germont, 76031 Rouen cedex, France; 11 Service de Réanimation Néonatale Pédiatrique, Hôpital Trousseau, Assistance Publique-Hôpitaux de Paris, 26 Avenue du Dr Arnold Netter, 75571 Paris, France; 12 Université Claude Bernard, Lyon 1, 8 Avenue Rockefeller, 69008 Lyon, France

**Keywords:** Malignant pertussis, Pediatric intensive care, Hyperleukocytosis, Leukodepletion, Risk factors of death

## Abstract

**Background:**

Malignant pertussis (MP) affects young infants and is characterized by respiratory distress, perpetual tachycardia and hyperleukocytosis up to 50 G/l, leading to multiple organ failure and death in 75% of cases. Leukodepletion may improve prognosis. A therapeutic strategy based on leukodepletion and extracorporeal life support (ECLS) according to different thresholds of leucocytes has been proposed by Rowlands and colleagues. We aimed at identifying factors associated with death and assess whether the respect of the Rowlands’ strategy is associated with survival.

**Methods:**

We reviewed all MP infants hospitalized in eight French pediatric intensive care units from January 2008 to November 2013. All infants younger than 3 months of age, admitted for respiratory distress with a diagnosis of pertussis and WBC count ≥ 50 G/l were recorded. Evolution of WBC was analyzed and an optimal threshold for WBC growth was obtained using the ROC-curve method. Clinical and biological characteristics of survivors and non-survivors were compared. Therapeutic management (leukodepletion and/or ECLS) was retrospectively assessed for compliance with Rowlands’ algorithm (indication and timing of specific treatments).

**Results:**

Twenty-three infants were included. Nine of 23 (40%) died: they presented more frequently cardiovascular failure (100% vs 36%, *p* = 0.003) and pulmonary hypertension (PHT; 100% vs 29%, *p* = 0.002) than survivors and the median [IQR] WBC growth was significantly faster among them (21.3 [9.7–28] G/l/day vs 5.9 [3.0–6.8] G/l/day, *p* = 0.007).

WBC growth rate > 12 G/l/day and lymphocyte/neutrophil ratio < 1 were significantly associated with death (*p* = 0.001 and *p* = 0.003, respectively).

Ten infants (43%) underwent leukodepletion, and seven (30%) underwent ECLS. Management following Rowlands’ strategy was associated with survival (100% vs 0%; *p* < 0.001, relative risk of death = 0.18, 95%-CI [0.05–0.64]).

**Conclusions:**

A fast leukocyte growth and leukocytosis with neutrophil predominance during acute pertussis infection were associated with death. These findings should prompt clinicians to closely monitor white blood cells in order to early identify infants at risk of fatal outcome during the course of malignant pertussis. Such an early signal in infants at high risk of death would increase feasibility of compliant care to Rowlands’ strategy, with the expectation of a better survival.

## Introduction

Pertussis is one of the leading causes of bacterial infection-related death in pediatric intensive care units (PICU) in France for young infants [1]. Severe pertussis encompasses two different groups of patients hospitalized in PICU; those of the first group present recurrent apnea or apparent life-threatening events, and those of the second bronchopneumonia, some of whom will develop a critical course with refractory hypoxemia and pulmonary hypertension (PHT) [2]. The latter clinical form, termed “malignant pertussis” (MP) was first described in 1956 [3]. MP affects young infants before 3 months of age, and is characterized by an acute respiratory distress, associated with perpetual tachycardia and hyperleukocytosis up to 50 G/l leading to multiple organ failure [4, 5]. Despite resuscitation and critical care, death occurs in up to 75% of cases [2, 4–9]. PHT and high white blood cell (WBC) count have been associated with a fatal outcome [2, 6, 7, 10–13]. Several “toxin-mediated” pathways are synergistic to explain fatalities: direct neurotoxicity, destruction of the respiratory epithelium inducing severe pneumonia, and profound systemic effects such as hyperleukocytosis by increasing circulating leukocyte mass [12–14]. Consequent hyper-viscosity result in arteriolar thrombosis with PHT, leading to cardiovascular failure and refractory hypoxemia [13, 15, 16]. These mechanisms were supported by post-mortem examination of lungs [13, 17] which led some physicians to propose leukodepletion (LD) with or without extracorporeal life support (ECLS) as adjunctive therapy to reduce this hyper-viscosity [18, 19], to decrease PHT, and to improve oxygenation [10], and therefore the prognosis. Murray et al*.* [11, 20] have demonstrated that infants with more severe illness had a more rapid rise in their total WBC count, suggesting that early WBC count monitoring would be useful to identify patients with poor outcome. Rowlands et al*.* [21] have proposed a therapeutic strategy based on cardiorespiratory failure (CRF) and WBC absolute count thresholds (50 G/l, 70 G/l and 100 G/l), reinforcing the importance of WBC close monitoring to manage the disease.

The present study therefore aimed to identify factors associated with death in infants affected by MP, from the time of admission to the emergency department (ED) and up to 24 h after admission to the PICU. We hypothesized that a rapid WBC growth is associated with a more severe form and a high risk of death. We further aimed to determine whether management according to Rowlands’ strategy is associated with survival.

## Material and methods

### Design and setting

A historical cohort study was conducted in eight French PICUs: Lyon, Marseille Hôpital Nord, Marseille la Timone, Montpellier, Grenoble, Saint-Etienne, Clermont-Ferrand, and Rouen. All infants younger than 3 months of age with positive RT-PCR for *Bordetella pertussis*, and admitted to a PICU for respiratory distress with hyperleukocytosis greater than 50 G/l, at any stage of the disease, were included. Cases were identified from the PICUs database all MP cases hospitalized from January 2008 to November 2013 were retrospectively reviewed. Clinical, biological, and imaging data were collected in a retrospective review, and recorded anonymously. Approval from the regional review board was obtained (Comité de Protection des Personnes, number QH 3/2016) and the requirement for informed consent was waived.

### Variables recorded and definitions

Demographic data, neonatal and postnatal history of the infant, season of the pertussis occurrence, vaccination status of the infant, and source of infection were collected. Clinical, biochemical, and hematological data were chronologically recorded during ED stay, at the time of transfer/admission to PICU, and during PICU stay. Worst values during the overall hospital stay were collected to characterize evolution of the disease. PHT was assessed on echocardiography findings. PHT was defined as mean pulmonary arterial pressure (mPAP) over 25 mmHg estimated by using either the measure of tricuspid regurgitation velocity or pulmonary regurgitation velocity. The PHT was judged as severe when it was associated with echocardiographic signs of right ventricular failure or if the mPAP was iso- or supra-systemic. The pediatric logistic organ dysfunction (PELOD) score was used to assess severity at PICU admission. SpO_2_/FiO_2_ ratio was used as a proxy for the PaO_2_/FiO_2_ ratio [22] in order to assess the severity of the lung injury, and acute respiratory distress syndrome (ARDS) was defined according to the pediatric acute lung injury consensus conference (PALICC) definition. Organ failures were described according to the Goldstein classification [23]. Cardio-respiratory failure was defined as the association of respiratory failure according to Goldstein criterion with signs of cardiovascular failure according to Goldstein criterion or with signs of cardiac failure on echocardiography. The cardiorespiratory failure was judged severe when it required invasive ventilation and vaso-inotrope support.

C-reactive protein (CRP), and when available procalcitonin (PCT), viral co-infection and bacterial superinfection were recorded.

We defined WBC growth as the absolute difference in WBC count over time in G/l/day using the following formula: (WBC′ − WBC)/(T′ − T), where WBC is the first value at ED admission, WBC′ is the value at time of transfer to PICU (or within 24 h following admission), and (T′ − T) is the interval between two blood samples in days. The lymphocyte/neutrophil ratio was computed and when WBC counts were maximal during ED and PICU stay; but WBC counts were no more considered after initiation of any specific procedure. The proportion of patients who had a lymphocyte/neutrophil ratio < 1 was also recorded.

Treatment included antibiotics, intensive care drugs, and LD, i.e., exchange transfusion or leukopheresis, combined or not combined with ECLS.

Indications for specific interventions (LD and/or ECLS) were retrospectively assessed for compliance with Rowlands’ algorithm in terms of indication and timing of specific treatments [21]. Briefly, we retained the following indications derived from the algorithm proposed by Rowlands: in case of CRF refractory to medical treatment, ECMO + LD is indicated if WBC > 50 G/l, ECMO ± LD if WBC > 30 G/l, and standard respiratory ECMO for all other situations. In case of CRF responsive to medical treatment, urgent LD is indicated if WBC > 100 G/l, if the infant has cardiac and respiratory failure with WBC > 70 G/l, and if the infant has cardiac or respiratory failure and PHT with WBC > 50 G/l; LD is indicated if WBC > 50 G/l is associated with cardiac or respiratory deterioration; standard intensive care is indicated for all other situations. To assess the compliance according to the algorithm, the clinical and biological course of the disease was submitted independently to two expert physicians (YH and RP), blinded to the binary outcome (“non-survivor” or “survivor”). The experts had to categorize the indication according to Rowlands’ algorithm [21]. Compliance or non-compliance were judged according to two criteria: (1) the realization of all indicated interventions, as any miss was considered as a deviation, and (2) interpreting the delay for the specific intervention: a delay > 24 h was considered as a deviation for any ECMO indication, or for any of the three LD indications flagged as “urgent”.

### Statistical analysis

Quantitative variables were reported as median and interquartile range [IQR]. Qualitative values were expressed as counts and percentages (%). Percentages were compared using Fisher’s exact test and distributions were compared using the Mann–Whitney *U* test. A result was considered as significant if the probability of the random occurrence was less than 5% (*p* < 0.05). WBC growth was studied using the receiver operating characteristic (ROC) method to test and separate the two groups with a threshold; The optimal cut-off value was defined as the value whose sensitivity and specificity were the closest to the value of the area under the ROC curve, and for which the absolute value of the difference between the sensitivity and specificity values was the smallest [24]. Agreement between experts was analyzed using Cohen’s Kappa coefficient and interpreted according to Landis and Koch [25].

All statistical analyses were performed using IBM SPSS statistics^®^ version 20.0 (IBM Armonk, NY, US).

## Results

A total of 23 infants with positive RT-PCR for *Bordetella pertussis*, and criteria for MP were identified in the eight PICUs between 2008 and 2013 (Table 1). All infants received targeted macrolide treatment. Source of infection was identified in 18 cases (78%): all contaminators were family members. Nineteen infants were not protected by vaccination: 17 (65%) were not vaccinated; two infants received only 1 dose (data not available for the four remaining cases).

**Table 1:** Patient characteristics at the time of emergency department admission and at PICU admission

	Non-survivors (*n* = 9)	Survivors (*n* = 14)	*p*	All patients (*n* = 23)
A: At emergency department admission				
Girl, *n* (%)	5 (56)	6 (43)	0.7	11 (48)
Postmenstrual age, weeks, median [IQR]	37 [36–39]	38 [35–40]	0.6	38 [36–40]
Preterm, *n* (%)				
< 37 weeks	2 (22)	5 (36)	0.6	7 (30)
< 33 weeks	0 (0)	2 (14)	0.5	2 (9)
Birth weight, kg, median [IQR]	2.5 [2.3–3.0]	2.9 [2.6–3.6]	0.1	2.9 [2.5–3.5]
Age, days, median [IQR]	31 [20–54]	57 [32–74]	0.1	41 [28–69]
Weight, kg, median [IQR]	3.5[2.9–4.0]	3.8 [3.1–5.9]	0.2	3.6 [3–4.6]
Heart rate, bpm, median [IQR]	176 [164–195]	169 [155–179]	0.3	170 [161–195]
Respiratory failure, *n* (%)	8 (89)	12 (86)	0.6	20 (87)
Sodium level, mmol/l, median [IQR]	138 [137–139]	135 [134–138]	0.1	137 [135–139]
C-reactive protein, mg, median [IQR]	67 [20–160]	4 [0–18]	0.02	10 [0–51]
pH, median [IQR]	7.25 [7.19–7.32]	7.32 [7.25–7.35]	0.1	7.29 [7.23–7.33]
pCO_2_, kPa, median [IQR]	8.2 [7.0–10.5]	7.4 [6.4–8.5]	0.4	7.5 [7.0–9.0]
WBC count, G/l, median [IQR]	26 [19–50]	36 [20–58]	0.6	32 [19–55]
Neutrophil count, G/l, median [IQR]	8 [6–14]	7 [5–13]	0.6	8 [5–13]
Lymphocyte count, G/l, median [IQR]	17 [10–30]	23 [12–39]	0.5	22 [10–37]
Lymphocyte/neutrophil ratio < 1, *n* (%)	1 (11)	1 (7)	1	2 (9)
B: At the time of transfer/admission to PICU				
PELOD score, median [IQR]	11 [10–20]	10 [1–14]	0.25	11 [1–20]
Heart rate, bpm, median [IQR]	193 [181–202]	179 [171–199]	0.25	185 [174–200]
Need for emergency intubation before PICU admission, *n* (%)	3 (33)	3 (21)	0.44	6 (26)
SpO_2_/FiO_2_ ratio, median [IQR]	426 [259–452]	384 [227–452]	0.87	384 [238–452]
pH, median [IQR]	7.29 [7.22–7.35]	7.31 [7.22–7.36]	0.51	7.30 [7.23–7.36]
pCO_2_ kPa, median [IQR]	7.3 [6.4–9.4]	7.19 [6.34–8.5]	0.68	7.3 [6.4–9.0]
Sodium level, mmol/l, median [IQR]	129 [126–135]	135 [131–136]	0.07	134 [128–136]
Lactate, mmol/l, median [IQR]	2 [2–4]	2 [1–3]	0.29	2 [1–3]
WBC count, G/l, median [IQR]	52 [38–91]	54 [41–60]	0.6	53 [42–81]

*IQR* interquartile range; *pCO*_*2*_ partial pressure of carbon dioxide; *PELOD* pediatric logistic organ dysfunction; *SpO*_*2*_*/FiO*_*2*_* ratio* pulse oximetry saturation/fraction of inspiratory oxygen; *WBC* white blood cell

A total of 14 infants survived, and 9 (39%) died after a median [IQR] delay since ED admission of 7 [4–12] days. Among nine non-survivors, seven infants died in the context of severe multiorgan failure combined in four of them with severe PHT; two infants died in the context of severe acute CRF subsequently put on ECLS with further complication of the procedure.

Broad-spectrum antibiotics were administered to 19 infants (83%) for suspected bacterial superinfection. Bacteria were identified in 14 infants, including six non-survivors (43%). Among the nine cases infected with enterobacteria or *S. pneumonia,* six were non-survivors (67%)*.* Nine infants presented viral co-infection, including four non-survivors. Four infants (17%) presented neither superinfection nor viral co-infection, and all survived. Compared to survivors, non-survivors presented more cardiovascular failure (89% vs 21%, *p* = 0.003), more PHT (100% vs 29%, *p* = 0.002), and more kidney failure (100% vs 36%, *p* = 0.003) (Table 2). During PICU stay, 17 infants (74%) were intubated and 13 (56%) required high frequency oscillation. Specific treatments included LD (exchange transfusion in eight, leukopheresis in two), and ECLS in seven infants (Table 3).

**Table 2:** Patient characteristics during PICU stay according to outcome

	Non-survivors (*n* = 9)	Survivors (*n* = 14)	*p*	All patients (*n* = 23)
Cardiovascular failure, *n* (%)	9 (100)	5 (36)	0.003	14 (61)
Pulmonary hypertension, *n* (%)	9 (100)	4 (29)	0.002	13 (56)
Kidney failure, *n* (%)	8 (89)	3 (21)	0.003	11 (48)
Anuria, *n* (%)	9 (100)	3 (21)	< 0.001	12 (52)
Neurological failure, *n* (%)	3 (33)	6 (43)	1	9 (39)
Liver failure, *n* (%)	5 (56)	2 (14)	0.049	7 (30)
Documented bacterial superinfection, *n* (%)^a^	6 (67)	8 (57)	1	14 (61)
Documented viral co-infection, *n* (%)^b^	4 (44)	5 (36)	1	9 (39)
Hematological failure, *n* (%)	5 (56)	2 (14)	0.02	7 (30)
Secondary thrombopenia, *n* (%)	7 (78)	6 (43)	0.19	13 (57)
Minimal sodium level, mmol/l, median [IQR]	122 [119–125]	131 [129–135]	0.003	130 [122–135]
Maximal lactate, mmol/l, median [IQR]	9 [3–16]	3 [2–4]	0.02	3 [2–9]
Minimal pH, median [IQR]	7.05 [6.8–7.1]	7.24 [7.17–7.32]	0.002	7.18 [7.05–7.26]
Maximal pCO_2_, kPa, median [IQR]	9.1 [8.7–12.3]	8.3 [7.0–11.0]	0.25	9.0 [7.7–11.5]
Maximal creatinine, µmol/l, median [IQR]	81 [49–110]	27 [23–51]	0.002	40 [25–82]
Maximal C-reactive protein, mg/l, median [IQR]	203 [135–237]	90 [7–205]	0.11	135 [30–217]
Maximal procalcitonin, µg/l, median [IQR]	19.0 [6.3–23.7]	1.0 [0.2–4.3]	0.012	3.3 [0.5–19.3]
WBC growth, G/l/day, median [IQR]	21 [10–28]	6 [3–7]	0.007	6 [3–17]
Maximal WBC count, G/l, median [IQR]	88 [63–95]	65 [53–81]	0.12	71 [56–91]
Maximal neutrophils, G/l, median [IQR]	41 [23–47]	25 [16–37]	0.14	26 [18–46]
Maximal lymphocytes, G/l, median [IQR]	33 [26–34]	33 [28–39]	0.83	32 [28–37]
Lymphocyte/neutrophil ratio < 1, *n* (%)	8 (89)	3 (21)	0.003	12 (52)

^a^*Stenotrophomonas maltophilia* (1), SAMS (4), *Escherichia coli* (2), *Klebsiella pneumoniae* + *Citrobacter koseri (1), Streptococcus pneumoniae* (2), *Enterobacter cloacae* (1), other GNB (1), *Haemophilus influenzae* (1), *Corynebacterium* (1) were identified through tracheal (1), nasopharyngeal (7), blood culture (5), and bronchoalveolar lavage (1) samples

^b^Rhinovirus (3), Picornavirus (3), Bocavirus (1), Influenza B (1), RSV (3), were identified through nasopharyngeal samples (8)

*IQR*  interquartile range; *pCO*_*2*_ partial pressure of carbon dioxide; *PELOD* pediatric logistic organ dysfunction; *PICU* pediatric intensive care unit; *SpO*_*2*_*/FiO*_*2*_* ratio* pulse oximetry saturation/fraction of inspiratory oxygen; *WBC* white blood cell

**Table 3:** Therapeutics used during PICU stay, and compliance with Rowlands’ algorithm [20]

	Non-survivors (*n* = 9)	Survivors (*n* = 14)	*p*	All patients (*n* = 23)
Invasive ventilation, *n* (%)	9 (100)	12 (86)	0.048	21 (91)
Treatment of pulmonary hypertension, *n* (%)				
Inhaled nitric oxide (INO)	6 (67)	3 (21)	0.07	9 (39)
Sildenafil as an add-on to INO	1 (11)	2 (14)	1	3 (13)
Renal replacement therapy, *n* (%)	4 (44)	2 (14)	0.16	6 (26)
Corticosteroids, *n* (%)	1 (11)	5 (36)	0.34	6 (26)
Broad-spectrum antibiotics	9 (100)	10 (71)	0.13	19 (83)
Leukodepletion, *n* (%)	3 (33)	7 (50)	0.7	10 (43)
Exchange transfusion, *n* (%)	2 (22)	6 (42)		8 (35)
Leukopheresis, *n* (%)	1 (11)	1 (7)		2 (8)
ECLS, *n* (%)	6 (67)	1 (7)	0.05	7 (30)
Compliance with Rowlands’ algorithm, *n* (%)	0 (0)	12 (86)	< 0.001	12 (52)

*ECLS* extracorporeal life support; *PICU* pediatric intensive care unit

## In the emergency department

At ED admission, median [IQR] age was 41 [28–69] days, and median [IQR] weight was 3.6 [3–4.6] kg. Twenty infants (87%) presented with respiratory distress (Table 1A) and 14 (61%) received oxygen during ED stay. Median CRP was 10 [0–51], median [IQR] WBC count at ED admission was 32 G/l [19–55]; lymphocyte/neutrophil ratio was ≥ 1 in 21 infants, and < 1 in two infants (Table 1A). Chest X-ray was performed in the ED for 22 infants, and found patchy infiltrates (*n* = 13), lobar infiltrates (*n* = 11), and/or atelectasis (*n* = 9); 4 were normal. All parameters were similar according to survival groups, except CRP which was significantly higher in non-survivors (67 vs 4 mg/l, *p* = 0.02).

At the time of transfer to the PICU, all children presented with perpetual tachycardia, and heavy lymphocytosis; PELOD, pH, WBC and lymphocyte counts, and need for emergency intubation before PICU admission were similar in both groups (Table 1B).

## In the pediatric intensive care unit

During PICU stay, median nadir of plasma sodium concentration was significantly lower in the non-survivors (122 vs 131 mmol/l; *p* = 0.003). Median maximal PCT value was 19.0 µg/l [6.3–23.7] in 5 non-survivors vs 1.0 µg/l [0.2–4.3] in 9 survivors (*p* = 0.012). Median WBC growth was significantly faster among the non-survivors compared to the survivors (21 vs 6 G/l/day, *p* = 0.007). A lymphocyte/neutrophil ratio < 1 was found in 11 (48%) infants, and was more frequently found in non-survivors than in survivors (89% *vs* 21%, *p* = 0.003) (Table 2).

**Fig. 1:**
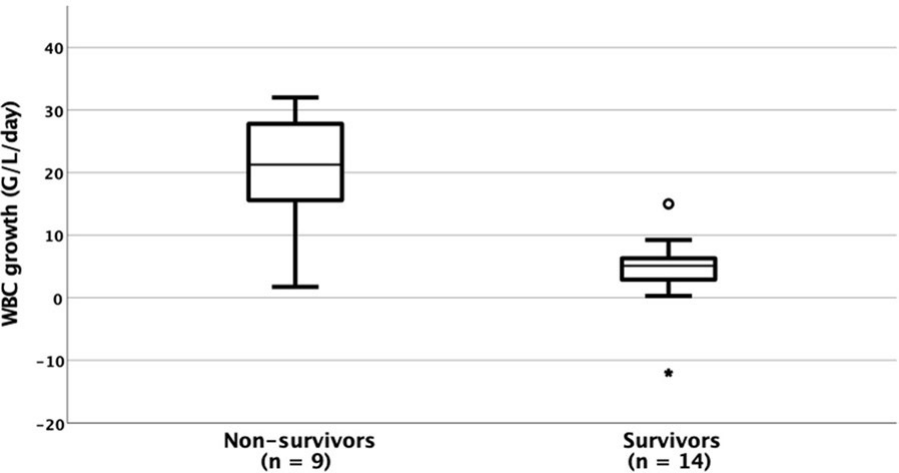
Boxplot of WBC growth according to the patient outcome. Box and whiskers plot quartiles and the bold band inside the box is the median. The ends of the whiskers represent the minimum and maximum of all of the data, with the exception of the two outliers for the survivors’ group, an open circle for the value over the [Q3 + 1.5 * (interquartile range)], and an asterisk for the value under the [Q1 − 3 * (interquartile range)]

Box plot of WBC growth shows unbalanced distribution according to survival groups (Fig. 1). ROC curve analysis indicated that WBC growth was strongly associated with death: the area under the curve (AUC) was 0.849 (95%CI [0.653–1.000]; *p* = 0.006), and the optimal cut-off value was obtained for a WBC growth of 12 G/l/day with a sensitivity of 78% and specificity of 93%. Eight infants (35%) had a WBC growth > 12 G/l/day. Infants with WBC growth > 12 G/l/day had significantly more frequently anuria (87% vs 33%, *p* = 0.03), had more frequently lymphocyte/neutrophil ratio < 1 (87% vs 27%; *p* = 0.009).

Treatment included invasive ventilation in 91%, LD in 43%, mostly by exchange transfusion (8/10), and ECLS in 30% (Table 3).

Indications according to Rowlands’ algorithm consisted of an urgent intervention (urgent LD and/or ECMO) in 16 infants (70%), the remaining consisted of three cases of non-urgent LD (13%), and four cases of standard intensive care (17%). Among the 11 cases of non-compliance to Rowlands’ algorithm, nine died; for seven cases, a delay in the diagnosis of MP and/or on the indication of LD and/or ECLS was identified as a main cause of non-compliance, suggesting a lack of early recognition of the disease and its severity. In three of these seven cases, the lack of ECLS availability on site was a factor explaining the delay (cases 12, 17 and 22); in a further three cases, infants presented with such a severe picture (cases 2, 14 and 21) that the rapidity of worsening prevented the initiation of the specific intervention required. All of the latter three cases died and for two of these, ECLS was not available on site requiring a transfer to another center. Non-availability of ECLS on site was identified as the sole reason of deviation in one fatal case (case 9).

Thus, treatment was compliant in 12 cases (52%); regarding agreement between experts, Cohen’s Kappa coefficient was 0.91 (*p* < 0.001), a value considered “almost perfect” according to Landis and Koch interpretation [25].

Nineteen infants (83%) presented one or more factors contributing to severity, including SAE from an ECMO procedure (cases 4, 9, and 17, i.e., 13%: one limb ischemia, one thrombosis of the ECMO circuit, and one failure of the ECMO procedure); neither AE nor SAE were reported for LD. A total of 9 infants (39%) were non-survivors (Table 4).

**Table 4:** Specific treatment indications, performed interventions, compliance to Rowlands’ algorithm, severity factors, and outcome

Indications according to [21]^b^	Performed interventions		Compliance to the algorithm		Factors contributing to severity	Outcome
	LD	ECLS	Yes/no	If no, criteria for deviation		
Urgent LD	2	0	Yes		PHT, KF, ARDS, seizures	Survivor
Urgent LD	1	0	No	> 24 h-delayed ECMO	Rapid worsening, ALF, KF, PHT, CPA	Non-survivor
VA-ECMO + LD	0	VA-ECMO		No combined LD	Viral co-infection	
Urgent LD	0	0	No	No LD	ALF, KF, ARDS, PHT	Non-survivor
VA-ECMO + LD	0	VV → VA-ECMO		> 24 h-delayed ECMO	Viral co-infection	
LD	0	0	No	No LD	Viral co-infection, PHT, ARDS	Non-survivor
VA-ECMO + LD	0	VV → VA-ECMO		> 24 h-delayed ECMO, no combined LD	ECMO complication: limb ischemia	
Urgent LD	1	0	Yes		None	Survivor
LD	1	0				
Urgent LD	1	0	Yes		Seizures	Survivor
LD	1	0	Yes		Bacterial superinfection, viral co-infection	Survivor
SIC	0	0	Yes		Viral co-infection	Survivor
VA-ECMO + LD	1	VA-ECMO	No	LD on time, but > 24 h-delayed ECMO + LD^a^	PHT, ARDS, KF, bacterial superinfections	Non-survivor
					ECMO complication: circuit thrombosis, extremities ischemia	
VA-ECMO	0	VA-ECMO	Yes		PHT, KF, bacterial superinfection	Survivor
SIC	0	0	Yes		None	Survivor
Urgent LD	0	0	No	No LD	ARDS, KF, CPA	Non-survivor
VA-ECMO + LD	0	0		No ECMO^a^, no combined LD		
Urgent LD	0	0	No	No LD	PHT, seizures	Survivor
					Bacterial superinfection	
VA-ECMO + LD	0	0	No	No ECMO^a^, no combined LD	Rapid worsening, ARDS, PHT, KF, bacterial superinfection	Non-survivor
LD	1	0	Yes		Bacterial superinfection, ARDS, HTP, KF	Survivor
Urgent LD	0	0	No	No LD	Viral co-infection, seizures	Survivor
Urgent LD	0	0	No	> 24 h-delayed LD	HTP, KF, ALF, CPA, seizures, viral co-infection	Non-survivor
VA-ECMO + LD	0	VA-ECMO		> 24 h-delayed ECMO^a^, no combined LD	ECMO complication: failure of ECMO procedure	
SIC	0	0	Yes		Bacterial superinfection, seizures, LTE	Survivor
SIC	0	0	Yes		None	Survivor
LD	1	0	Yes		None	Survivor
VA-ECMO + LD	0	0	No	No VA-ECMO^a^, no combined LD	Rapid worsening, ARDS, PHT, bacterial superinfection	Non-survivor
Urgent LD	1	0	No	> 24 h-delayed LD	ARDS, CPA, PHT, KF, ALF, seizures	Non-survivor
VA-ECMO + LD	0	VA-ECMO		> 24 h-delayed ECMO^a^, no combined LD	Bacterial superinfections	
Urgent LD	1	0	Yes		Bacterial superinfections*,* ARDS, seizures	Survivor

In case of refractory CRF: ECMO + LF if [WBC > 50], ECMO ± LF if [WBC > 30], and standard respiratory ECMO for all other situations

^a^ECLS not available on site: transfer to another center was required

^b^Indications refers to Rowlands’ algorithm [21]. In case of responsive CRF: urgent LD if [WBC > 100] or [WBC > 70 + CF + RF] or [WBC > 70 + (CF or RF) + PHT], LD if [WBC > 50 + (cardiac or respiratory deterioration)] and standard intensive care for all other situations

*ARDS* acute respiratory distress syndrome; *ALF* acute liver failure; *KF* kidney failure; *CPA* cardiopulmonary arrest; *ECLS* extracorporeal life support; *LD* leukodepletion; *LTE* life-threatening event; *PHT* pulmonary hypertension; *SIC* standard intensive care; *VA-ECMO* veno-arterial-extracorporeal membrane oxygenation; *VV-ECMO* veno-venous-ECMO

At the time of PICU admission, there was no significant difference between infants treated or not according to Rowlands’ algorithm, even in terms of median [IQR] PELOD score (11 [10–20] vs 10 [1–18], *p* = 0.26). All infants (100%, 8/8 of whom 7 non-survivors) having WBC growth > 12 G/day had a non-compliant treatment according to Rowlands’ algorithm compared to 20% (3/15 of whom 2 non-survivors) in those who had a WBC growth ≤ 12 G/day (*p* < 0.001, relative risk of non-compliance = 0.20 (95% CI [0.07–0.55]); all the 12 infants with WBC growth ≤ 12 G/day and compliant treatment were survivors.

## Discussion

In the present study, mortality in cases of MP was systematically associated with cardiovascular failure, PHT, and hyperleukocytosis. Moreover, WBC growth in non-survivors was three times faster than in those who survived, and a lymphocyte/neutrophil ratio < 1 before LD as well as a higher median CRP at ED admission were both associated with death. A close monitoring of WBC count, WBC growth, and lymphocyte/neutrophil ratio may help physicians to decide when to transfer infants to PICU and when to initiate invasive specific interventions such as LD and/or ECLS.

In line with that reported elsewhere, the present study found that hyperleukocytosis was associated with death [5–8, 10, 26, 27]. There was, however, some heterogeneity in the recruitment of patients and in definitions used for severe pertussis. It is of note that mortality found herein is coherent with the literature, being between that reported in studies recruiting from the total PICU population (ranging from 14 to 35% [2, 6, 10, 26]) and those investigating specifically high-risk groups (55% in patients with pneumonia, PHT, or receiving vasoactive drugs [4], 78% in patients with pneumonia [2], and 71–72% in those receiving ECLS [9, 28]). Malignant pertussis was defined herein as WBC count ≥ 50 G/l in infants < 3 months of age and it is noteworthy that WBC counts and severity parameters at PICU admission were not significantly different between survivors and non-survivors, which indicates that other factors should be explored to predict death. For instance, herein, WBC growth during PICU stay was significantly different between non-survivors and survivors; more specifically, WBC growth > 12 G/l/day was associated with PHT and death, whereas PELOD at PICU admission were similar between those with a growth ≤ 12 G/l/day and those with a growth > 12 G/l/day. This could suggest that WBC growth may precede clinical features and predict the severity of the disease more than a WBC count threshold. In addition, lymphocytosis is classically described as a consequence of pertussis infection [29], but for nearly 90% non-survivors neutrophils predominance occurred. This characteristic was also found by Ganeshalingham et al*.* [4] but the authors compared the subgroup of children with pneumonia, PHT, or receiving vasoactive drugs vs the others, without testing it against death. It may hypothesize that bacterial superinfection and/or severe inflammatory response associated with pertussis may increase neutrophil count. In line with this hypothesis, a high PCT level during PICU stay was associated with poor outcome (and there was also a trend towards this for CRP), and two-thirds non-survivors developed bacterial superinfection which is consistent with the recent suggestion made by Kazantzi et al. and Berger et al. [6, 30] that bacterial co-infection might contribute to the severity of illness and mortality. Another point of note is that all non-survivors developed PHT associated with respiratory and cardiovascular failure, which are well known factors associated with death [9, 12, 27]. This emphasizes the need for a systematic echocardiography at admission and during hospitalization to detect PHT and cardiac failure. Furthermore, all non-survivors developed anuria during their PICU stay and in such cases monitoring urine output and fluid overload estimation is crucial as it could lead to an earlier decision for renal replacement therapy.

Taken together, these parameters could be useful to identify early on infants who should be quickly transferred to a specialized PICU able to initiate LD and/or ECLS. In the present study, compliance with Rowlands’ algorithm was associated with an improved outcome, as all patients treated according to the algorithm survived. Furthermore, infants who were not treated in accordance with Rowlands’ algorithm were those who had the faster disease course. One of the possible explanations could be a late PICU admission in a more severe respiratory and/or hematological failure preventing the application of LD at the right time. Indeed, among the 11 infants who were not treated in accordance with Rowlands’ algorithm, seven were treated with a delay or did not receive specific interventions when indicated suggesting a non-recognition of the severity, whereas only three were identified as rapidly worsening not allowing timely interventions. Moreover, initial characteristics at PICU admission of infants who were not treated according to Rowlands’ algorithm did not differ significantly from those treated in accordance with the strategy—except for WBC count and WBC growth that were higher. All infants with WBC growth ≤ 12 G/l/day did receive a treatment compliant with Rowlands’ algorithm and survived, suggesting that assessment and monitoring was possible; conversely, when WBC growth was > 12 G/l/day, the clinical course may have prevented application of Rowlands’ algorithm. This result confirms the prognostic value of leukocytes. An early determination of WBC growth and its monitoring may help PICU physicians to decide to initiate invasive treatment such as LD in order to improve the outcome, as it was already suggested by Nieves et al*.* [31]. The availability of ECLS on site was identified in the present study as a potential cofactor of non-compliance to Rowlands’ algorithm contributing to a poor outcome. This result highlights the role of the orientation of infants at high risk of deterioration to a center where ECLS and LD are available, or the implementation of a mobile ECLS team able to put infants on ECMO and transfer them to the ECLS center. This may have implications in terms of regional organization of the health care system for infants with severe CRF secondary to pertussis infection.

This cohort is one of the largest reported and involved multiple centers in France. However, it is limited by the small sample size due to the rarity of the disease and its retrospective design; the results should be interpreted appropriately. In cases where infants were hospitalized in other hospitals before their PICU admission, previous biological analyses were not systematically done and consequently some data are missing. Rapidly evolving disease may explain how hard it is to define prognostic criteria. Protocol and therapeutic management—especially application of the Rowlands’ algorithm or availability of technical equipment (such as ECLS) may differ from one center to another, and this can create measurement bias. To test the hypothesis of a better outcome if LD was performed earlier, a randomized controlled trial should be conducted, but the rarity of MP makes it difficult.

## Conclusion

In infants with the diagnosis of severe pertussis, screening of inflammatory parameters, monitoring of leukocytes (not only absolute counts but growth per day) and lymphocyte/neutrophil ratio may help physicians to select early those requiring LD and/or ECLS. The consideration of these criteria along with the clinical course would increase compliance with Rowlands’ strategy, with the expectation of better survival.

## Data Availability

The datasets used and/or analyzed during the current study are available from the corresponding author on reasonable request.

## Abbreviations

ARDS: Acute respiratory distress syndrome
CRF: Cardio-respiratory failure
ECLS: Extracorporeal life support
ET: Exchange transfusion
ED: Emergency department
IQR: Interquartile range
KF: Kidney failure
LD: Leukodepletion
MP: Malignant pertussis
PELOD: Pediatric logistic organ dysfunction
PHT: Pulmonary hypertension
PICU: Pediatric intensive care unit
WBC: White blood cell
